# The association between self-reported and objective measures of health and aggregate annoyance scores toward wind turbine installations

**DOI:** 10.17269/s41997-018-0041-x

**Published:** 2018-04-12

**Authors:** David S. Michaud, Leonora Marro, James McNamee

**Affiliations:** 1 Health Canada, Environmental and Radiation Health Sciences Directorate, Consumer and Clinical Radiation Protection Bureau, Non-Ionizing Radiation Health Sciences Division, 775 Brookfield Road, Ottawa, ON K1A 1C1 Canada; 2 Health Canada, Environmental Health Science and Research Bureau, Population Studies Division, Biostatistics Section, Ottawa, ON K1A 0K9 Canada

**Keywords:** Noise, Principal component analysis, Community survey, Renewable energy, Canada, Bruit, Analyse en composantes principales, Enquête communautaire, Énergie renouvelable, Canada

## Abstract

**Objective:**

An aggregate annoyance construct has been developed to account for annoyance that ranges from *not at all annoyed* to *extremely annoyed*, toward multiple wind turbine features. The practical value associated with aggregate annoyance would be strengthened if it was related to health. The objective of the current paper was to assess the association between aggregate annoyance and multiple measures of health.

**Methods:**

The analysis was based on data originally collected as part of Health Canada’s Community Noise and Health Study (CNHS). One adult participant per dwelling (18–79 years), randomly selected from Ontario (ON) (*n* = 1011) and Prince Edward Island (PEI) (*n* = 227), completed an in-person questionnaire.

**Results:**

The average aggregate annoyance score for participants who indicated they had a health condition (e.g., chronic pain, Pittsburgh Sleep Quality Index (PSQI) > 5, tinnitus, migraines/headaches, dizziness, highly sensitive to noise, and reported a high sleep disturbance) ranged from 2.53 to 3.72; the mean score for those who did not report these same conditions ranged between 0.96 and 1.41. Household complaints about wind turbine noise had the highest average aggregate annoyance (8.02), compared to an average of 1.39 among those who did not complain.

**Conclusion:**

A mean aggregate annoyance score that could reliably distinguish participants who self-report health effects (or noise complaints) from those who do not could be one of several factors considered by jurisdictions responsible for decisions regarding wind turbine developments. However, the threshold value for acceptable changes and/or levels in aggregate annoyance has not yet been established and could be the focus of future research efforts.

## Résumé

**Objectif:**

Un indice de gêne global, de *pas du tout gênant* à *extrêmement gênant*, a été élaboré pour tenir compte de la gêne causée par de nombreuses caractéristiques des éoliennes. La valeur pratique associée à la gêne globale serait renforcée si celle-ci était liée à la santé. L’objectif était d’évaluer l’association entre la gêne globale et divers indicateurs de santé.

**Méthode:**

Cette analyse est fondée sur des données recueillies à l’origine dans le cadre de l’Étude sur le bruit ambiant et la santé (ÉBAS) de Santé Canada. Des participants adultes (18 à 79 ans), un par ménage, sélectionnés au hasard en Ontario (*n* = 1011) et à l’Île-du-Prince-Édouard (*n* = 227), ont rempli un questionnaire en personne.

**Résultats:**

En moyenne, l’indice de gêne global des participants ayant fait état d’une affection de santé (p. ex. douleur chronique, indice de qualité du sommeil de Pittsburgh [PSQI] >5, acouphène, migraines/maux de tête, étourdissements, forte sensibilité au bruit et perturbation élevée du sommeil) se situait entre 2,53 et 3,72; l’indice moyen des participants n’ayant pas déclaré ces mêmes affections se situait entre 0,96 et 1,41. Les plaintes des ménages au sujet du bruit des éoliennes ont été associées en moyenne à l’indice de gêne global le plus élevé, soit 8,02, contre 1,39 en moyenne chez les participants qui ne se plaignaient pas du bruit des éoliennes.

**Conclusion:**

Un indice de gêne global moyen permettant de façon fiable de distinguer les participants qui font état d’effets sur leur santé (ou qui se plaignent du bruit) de ceux qui ne déclarent pas de tels effets pourrait être l’un de plusieurs facteurs à considérer par les administrations qui prennent des décisions sur le développement éolien. Toutefois, le seuil de gêne globale acceptable (son niveau et/ou son changement) reste à définir et pourrait faire l’objet d’études futures.

## Introduction

An aggregate annoyance construct has been developed to account for magnitudes of annoyance that range from *not at all annoyed* to *extremely annoyed* toward five wind turbine features (Michaud et al. 2018). These features included noise, shadow flickers, blinking lights, visual impacts, and vibrations. The construct was developed in recognition of the observation that wind turbine noise (WTN) was not the only, nor the most prevalent, wind turbine feature associated with community annoyance in the Community Noise and Health Study (CNHS). An aggregate annoyance score provides a more comprehensive assessment of annoyance than can be gleaned from any individual feature in isolation. The setback distance that corresponds with a statistically significant change in an aggregate annoyance score can inform jurisdictions that set policy. Although the point of departure from the curve is informative, there may be added value in knowing if there is, on average, an aggregate annoyance score that can reliably distinguish groups reporting health effects from those that do not.

As discussed elsewhere (Michaud et al. 2018), principal component analysis (PCA) weights each annoyance response in terms of how much it contributes to the aggregate annoyance construct. However, the authors acknowledge that the validity of the construct as one that has relevance to health or well-being is based on the tacit assumption that the valuation of significance placed on the items that constitute aggregate annoyance is reflected in the magnitude of rated annoyance assigned to each by study participants. The science base available to date does not refute this assumption; however, as outlined in the “Discussion” section, evaluating an untested assumption of equivalence could be a focus of future research in this area.

Previous research has demonstrated a statistical association between *high* noise annoyance and several measures of reported and measured health outcomes (Basner et al. 2014; WHO 2011; Niemann et al. 2006), including several objectively measured outcomes in Health Canada’s CNHS (Michaud et al. 2016a). While statistical associations between high noise annoyance and some indicators of health are clearly insufficient to conclude a causal relation between annoyance and health, they may provide support for efforts that aim to mitigate long-term high noise annoyance. The same analysis has not yet been conducted for a measure that is based on several variables related to annoyance (i.e., aggregate annoyance).

Aggregate annoyance represents a novel approach to evaluating community annoyance. The adoption of this approach over conventional methods requires that there is a predictable change in aggregate annoyance as a function of proximity to wind turbines similar to that reported elsewhere (Michaud et al. 2018). Moreover, the pragmatic application of presenting an aggregate annoyance score as representing a community’s magnitude of total annoyance toward wind turbines would be more defensible if the aggregate annoyance score was shown to be statistically related to measures of health and/or well-being. To this end, the primary purpose of the current analysis was to assess the mean aggregate annoyance scores among participants’ health outcomes measured in the CNHS. The specific health measures assessed were based on their claimed attribution to WTN exposure (e.g., dizziness, tinnitus, migraines, sleep disturbance, depression) or the idea that they may be altered if annoyance represents or influences a stress response. Multiple measures of stress were reported and objectively measured in the CNHS, including but not limited to hair cortisol, blood pressure, heart rate, and perceived stress.(Michaud et al. 2016a)

## Methods

### Study characteristics

The current study is a secondary analysis of the data collected as part of Health Canada’s CNHS. Any duplication of the methods already presented is intentional and considered the minimum necessary for the current analysis to stand on its own. The study characteristics have been described in another publication (Michaud et al. 2016b). Briefly, dwellings were identified from two Canadian provinces. The ON and PEI sampling regions included 315 and 84 wind turbines and 1011 and 227 dwellings, respectively. The wind turbine electrical power outputs ranged between 660 kW and 3 MW (average 2.0 ± 0.4 MW). Turbine hub heights were predominantly 80 m. To maximize sampling in areas where potential impacts from WTN exposure would be most likely to occur, a “take-all” sampling strategy was employed for all identified dwellings within approximately 600 m of a wind turbine. The remaining dwellings were selected randomly up to approximately 11 km. From each dwelling, one participant between the ages of 18 and 79 years was randomly chosen to participate. No substitution was permitted under any circumstances, and participants were not compensated for their participation.

### Data collection

The full study questionnaire is available in the supplementary materials elsewhere (Michaud et al. 2016b). Statistics Canada-trained interviewers (16) conducted in-person home interviews between May 2013 and September 2013. In addition to basic demographic variables and previously validated content, the questionnaire’s *perception module* included several questions on annoyance to multiple wind turbine features. In addition to noise, participants were also asked to indicate their magnitude of annoyance toward turbine blinking lights, shadows or flickers of light, visual impacts, and vibration or rattles noticed indoors which coincided with a participant’s recollection of wind turbine operations. Annoyance response categories included *not at all*, *slightly*, *moderately*, *very*, and *extremely*. Pertinent to the current analysis, the questionnaire also included several health-related measures, including but not limited to, chronic pain, stress, blood pressure, tinnitus, migraines, dizziness, quality of life, and sleep disturbance. For brevity, methodological procedures for measured blood pressure, heart rate, and hair cortisol levels are presented elsewhere (Michaud et al. 2016c). In an attempt to mask the study’s focus on wind turbines, potential participants were informed that the purpose of the survey was to investigate the potential impact on health from community noise.

### Statistical methodology

#### Derivation of an aggregate annoyance construct

The method for deriving the aggregate annoyance construct has been reported elsewhere (Michaud et al. 2018). Briefly, a PCA was conducted in order to discover and summarize the pattern of intercorrelations among the five evaluated wind turbine features (i.e., “annoyance features”). The information derived from this preliminary investigation was then used to predict a single criterion variable for annoyance based on the five wind turbine features. Aggregate annoyance was based on all magnitudes of annoyance from not at all annoyed to extremely annoyed (0: not at all, 1: slightly annoyed, 2: moderately annoyed, 3: very annoyed, 4: extremely annoyed) and therefore reflects the combined annoyance toward multiple wind turbine features. The possible range in aggregate annoyance was 0 to 20. A score of 0 reflects no perception/annoyance toward any wind turbine feature and a score of 20 reflects extreme annoyance toward all 5 features.

#### Relationship between aggregate annoyance and health conditions

An ANOVA was performed based on the constructs derived from the PCA to compare aggregate annoyance levels with the presence or absence of self-reported health conditions. The variability due to distance and province were accounted for in the ANOVA models. The analysis was reanalyzed using A-weighted WTN categories in place of distance categories (see supplemental material). This was not repeated with C-weighted WTN levels as the results would essentially mirror A-weighted findings due to the high correlation between dBA and dBC values (i.e., Pearson’s linear correlation coefficient *r* > 0.8) (Keith et al. 2016). The assumptions of the ANOVA were verified using the Anderson-Darling test for normality and Levene’s test for equal variance of the residuals. When the assumptions were not satisfied, non-parametric methods were applied (i.e., the data were ranked, and the analysis was conducted on the ranks of the data). Self-reported variables of interest included chronic pain, high blood pressure, heart disease, quality of sleep, quality of life, satisfaction with one’s health, tinnitus, migraines/headaches, dizziness, medication for anxiety/depression, noise sensitivity, sleep disturbance, lodging a complaint about wind turbines, and reporting to receive personal benefits from having wind turbines in the area. Quality of sleep was based on the Pittsburgh Sleep Quality Index (PSQI) where values greater than 5 are considered to indicate “poor” sleep (Buysse et al. 1989). Quality of life and satisfaction with one’s health are based on the two stand-alone questions from the WHOQOL-BREF questionnaire (WHOQOL Group 1998). As reported elsewhere (Feder et al. 2015), participants were considered to have a poor quality of life if they responded either “poor” or “very poor” to *In the past month, how would you rate your quality of life?* All other responses (“neither poor nor good,” “good,” “very good”) were considered to indicate participants have a good quality of life. Similarly, participants were considered to be “dissatisfied” with their health if they responded either “dissatisfied” or “very dissatisfied” to *In the past month, how satisfied were you with your health?* All other responses (“neither dissatisfied nor satisfied”, “satisfied”, very satisfied) were considered to indicate participants were satisfied with their health (Feder et al. 2015). ANOVA models relating self-reported health conditions and aggregate annoyance were further adjusted for age and sex, in addition to distance to the nearest turbine and province. Spearman correlation coefficient and linear regression models were used to investigate the relationship between the overall annoyance construct and the following continuous variables: systolic blood pressure, diastolic blood pressure, heart rate, hair cortisol levels, perceived stress scale (PSS), PSQI, and the four WHOQOL-BREF domains (physical health, psychological well-being, social relationships, and environmental factors). Again, these linear regression models were adjusted for distance to nearest turbine and province, and then refit adjusting for age and sex in addition to distance to nearest turbine and province.

The data analysis for this paper was generated using SAS/STAT software, version 9.2 of the SAS System for Windows 7. Unless otherwise indicated, a 5% significance level (α = 0.05) was implemented throughout.

This study was approved by the Health Canada and Public Health Agency of Canada Review Ethics Board in accordance with the Tri-Council Policy Statement Ethical Conduct For Research Involving Humans (TCPS) (Protocol no. 2012-0065 and no. 2012-0072).

## Results

### Relationship between aggregate annoyance and health conditions

The association between aggregate annoyance (which reflects all levels of annoyance, from *not at all annoyed* to *extremely annoyed*) and self-reported health outcomes or other negative reactions to noise (e.g., complaints) was investigated. Table 1 presents the results when relating aggregate annoyance to various health conditions originally reported in Health Canada’s CNHS (Michaud et al. 2016b). Self-reported variables of interest in the current analysis included chronic pain, high blood pressure, heart disease, quality of sleep, quality of life, satisfaction with one’s health, tinnitus, migraines/headaches, dizziness, medication for anxiety/depression, noise sensitivity, and sleep disturbance. In addition, lodging a complaint about noise from wind turbines and reporting to receive personal benefit from having wind turbines in the area were assessed.

**Table 1:** Aggregated annoyance related to specific outcome assessed

Variable	Number	ANOVA model adjusted for distance and province^a^		ANOVA model adjusted for distance, province, age, and sex^b^	
		Least squares means (95% CI)^c^	*p* value^d^	Least squares means (95% CI)^e^	*p* value^d^
Sex					
Male	600	1.89 (1.47, 2.31)	0.0345		
Female	626	1.46 (1.05, 1.87)			
Age group (years)					
≤24	72	0.63 (−0.29, 1.54)	0.0089		
25–44	327	1.65 (1.16, 2.14)			
45–64	543	1.94 (1.51, 2.37)			
65+	284	1.38 (0.85, 1.91)			
Chronic pain					
Yes	285	2.69 (2.16, 3.22)	0.0001	2.47 (1.89, 3.05)	0.0002
No	939	1.41 (1.04, 1.78)		1.20 (0.80, 1.61)	
High blood pressure					
Yes	368	1.52 (1.04, 2.01)	0.3909	1.20 (0.65, 1.76)	0.1962
No	854	1.72 (1.34, 2.10)		1.48 (1.06, 1.90)	
Heart disease					
Yes	94	1.45 (0.63, 2.26)	0.3341	1.15 (0.31, 2.00)	0.2533
No	1131	1.68 (1.32, 2.04)		1.42 (1.02, 1.83)	
Reported “poor” sleep					
PSQI > 5	549	2.53 (2.11, 2.96)	< 0.0001	2.31 (1.84, 2.77)	< 0.0001
PSQI ≤ 5	650	0.96 (0.54, 1.37)		0.75 (0.31, 1.19)	
Rated QOL, previous month^f^					
Poor	80	2.41 (1.54, 3.28)	0.1187	2.14 (1.25, 3.02)	0.1372
Good	1144	1.61 (1.25, 1.98)		1.36 (0.95, 1.76)	
Rated satisfaction with health, previous month^g^					
Dissatisfied	173	2.32 (1.69, 2.95)	0.1086	2.04 (1.38, 2.70)	0.1392
Satisfied	1053	1.56 (1.19, 1.93)		1.31 (0.91, 1.72)	
Tinnitus					
Yes	290	2.89 (2.38, 3.40)	< 0.0001	2.63 (2.09, 3.17)	< 0.0001
No	935	1.28 (0.91, 1.65)		1.02 (0.61, 1.43)	
Migraines^h^					
Yes	287	3.49 (2.98, 4.01)	< 0.0001	3.37 (2.83, 3.92)	< 0.0001
No	938	1.21 (0.85, 1.57)		0.90 (0.50, 1.29)	
Dizziness					
Yes	270	3.00 (2.48, 3.53)	< 0.0001	2.82 (2.26, 3.37)	< 0.0001
No	956	1.30 (0.94, 1.67)		1.04 (0.63, 1.45)	
Medication for anxiety or depression					
Yes	141	1.51 (0.83, 2.20)	0.2415	1.30 (0.59, 2.02)	0.3293
No	1085	1.68 (1.31, 2.05)		1.41 (1.01, 1.82)	
Noise sensitivity^i^					
High	171	3.72 (3.10, 4.34)	< 0.0001	3.52 (2.87, 4.18)	< 0.0001
Less than high	1051	1.36 (1.00, 1.72)		1.14 (0.74, 1.54)	
Long-term sleep disturbance^j^					
High	162	3.48 (2.84, 4.12)	< 0.0001	3.25 (2.58, 3.93)	< 0.0001
Less than high	1061	1.41 (1.05, 1.77)		1.19 (0.79, 1.59)	
Household complaint lodged regarding WTN					
Yes	34	8.02 (6.79, 9.24)	< 0.0001	7.73 (6.48, 8.97)	< 0.0001
No	1189	1.39 (1.04, 1.74)		1.18 (0.79, 1.56)	
Personal benefit^k^					
Yes	110	− 0.11 (− 0.88, 0.66)	< 0.0001	− 0.36 (− 1.15, 0.43)	< 0.0001
No	1064	1.93 (1.54, 2.31)		1.68 (1.25, 2.11)	

^a^Analysis of variance (ANOVA) model of aggregate annoyance related to variable, model adjusted for province and distance to turbines

^b^ANOVA model of aggregate annoyance related to variable, model adjusted for province, distance to turbines, age, and sex

^c^Least squares means of aggregate annoyance and corresponding 95% confidence interval (CI) after adjusting for province and distance to turbines

^d^*p* values are based on the ranks of the data (non-parametric statistics)

^e^Least squares means of aggregate annoyance and corresponding 95% confidence interval (CI) after adjusting for province, distance to turbines, age, and sex

^f^Poor includes ratings of “poor” and “very poor”; good includes ratings “neither poor nor good,” “good,” and “very good”

^g^Dissatisfied includes the ratings “dissatisfied” and “very dissatisfied”; satisfied includes the ratings “neither satisfied or dissatisfied,” “satisfied,” and “very satisfied”

^h^Frequent migraines or headaches (includes nausea, vomiting, sensitivity to light and sound)

^i^Noise sensitivity was defined as “high” for participants who reported to be very or extremely sensitive and “less than high” for participants who reported to be not at all, slightly, or moderately sensitive

^j^The magnitude of reported sleep disturbance over the previous year while at home for any reason was defined as “high” for participants who reported to be very or extremely sleep disturbed and “less than high” for participants who reported to be not at all, slightly or moderately sleep disturbed

^k^Includes benefit through rent, payments, or other indirect benefits such as a hall or community centre for having wind turbines in their area

All health conditions were equally distributed between distance groups and dBA WTN groups (results not shown). Least squares means and confidence intervals were based on the mean of the total five annoyance features for each participant; *p* values of the models were based on non-parametric statistics of the first construct from PCA for the overall annoyance. A significant increase in average aggregate annoyance was observed among participants who self-reported to have chronic pain, scores on the PSQI above 5 (i.e., poor sleep), tinnitus, migraines/headaches, dizziness, reported very or extreme (i.e., high) sensitivity to noise, and reported very or extreme (i.e., high) sleep disturbance at home over the last year, for any reason.

An increase in average aggregate annoyance was also observed among those who lodged a complaint as well as among those who did not receive personal benefits. Age and sex were also related to aggregate annoyance; participants between the ages of 45 and 64 years had higher aggregate annoyance scores when compared to other age categories, as did males compared to females. Further adjusting the models for age and sex differences did not affect the results (see Table 1). For the self-reported health variables considered, the average aggregate annoyance score for those participants who indicated they had a health condition (e.g., chronic pain, PSQI > 5, tinnitus, migraines/headaches, dizziness, highly sensitive to noise, and reported a high sleep disturbance) ranged from 2.53 to 3.72; the mean aggregate annoyance for those who did not exhibit these same health conditions ranged between 0.96 and 1.41. Participants who reported that someone in their household lodged a formal complaint (34 participants) had the highest average aggregate annoyance (i.e., 8.02), compared to an average of 1.39 among those who did not lodge a formal complaint. Aggregate annoyance was effectively 0 (i.e., least squares mean − 0.11) among the 110 participants who reported to receive personal benefit from having wind turbines in the area, compared to an average of 1.93 among those who did not report such benefits.

Similar results were detected when the analysis was conducted with A-weighted WTN levels (see supplemental material). For example when A-weighted WTN levels were used in place of proximity to turbines, a significant increase in average aggregate annoyance was also observed among participants who self-reported to have chronic pain, scores on the PSQI above 5 (i.e., poor sleep), tinnitus, migraines/headaches, dizziness, reported very or extreme (i.e., high) sensitivity to noise, and reported very or extreme (i.e., high) sleep disturbance at home over the last year, for any reason. Again, the average aggregate annoyance score for those participants who reported these health effects ranged from 2.38–3.50; the mean aggregate annoyance for those who did not report these same health conditions ranged from 0.78 to 1.27.

Finally, linear regression models, after adjustments were made for age and sex, revealed that diastolic blood pressure, PSS, and PSQI scores were positively associated with increased values of aggregate annoyance (see Table 2). For example, for every unit increase in the log-transformed diastolic blood pressure (log mmHg), aggregate annoyance would increase by 2.28 (SE 0.86, *p* = 0.0084). Aggregate annoyance would increase by 0.07 (SE 0.02, *p* < 0.0001) for every unit increase in PSS and by 0.21 (SE 0.03, *p* < 0.0001) for every unit increase in PSQI. From the WHOQOL-BREF, physical health, psychological well-being, and environmental factors domains were negatively associated with increased values of aggregate annoyance (see Table 2). Larger domain values indicate a healthier QOL for the respective domain. For example, as physical health domain increased, aggregate annoyance decreased by − 0.23 (SE 0.04, *p* < 0.0001); as the psychological well-being index increased, aggregate annoyance decreased by − 0.12 (SE 0.04, *p* = 0.0085); as the environmental factors index increased, aggregate annoyance decreased by − 0.25 (SE 0.05, *p* < 0.0001). All model-adjusted *R*^2^ ranged between 7% and 12%. Results were similar when A-weighted WTN levels were used in the linear regression model (see supplemental material).

**Table 2:** Aggregated annoyance related to specific health condition, continuous variables

Variable (minimum, maximum)	Number	Spearman correlation coefficient (*p* value)	Adjusted *R*^2^ of the linear regression model^a^	Linear regression of aggregate annoyance relative to the variable^a^		Adjusted *R*^2^ of the linear regression model^c^	Linear regression of aggregate annoyance relative to the variable^c^	
				Slope (SE)^b^	*p* value		Slope(SE)^b^	*p* value
Systolic blood pressure (83, 186)	1066	0.06 (0.0580)	0.07	0.01 (0.01)	0.0911	0.07	0.01 (0.01)	0.1356
log(systolic blood pressure) (4.42, 5.23)	1066	0.06 (0.0580)	0.07	1.54 (0.84)	0.0682	0.07	1.48 (0.91)	0.1041
Diastolic blood pressure (50, 114)	1066	0.12 (0.0001)	0.08	0.03 (0.01)	0.0066	0.08	0.03 (0.01)	0.0118
log(diastolic blood pressure) (3.91, 4.74)	1066	0.12 (0.0001)	0.08	2.41 (0.85)	0.0047	0.08	2.28 (0.86)	0.0084
Heart rate (41, 125)	1066	0.02 (0.4222)	0.07	0.00 (0.01)	0.7764	0.07	0.00 (0.01)	0.8553
log(heart rate) (3.71, 4.83)	1066	0.02 (0.4222)	0.07	− 0.15 (0.70)	0.8301	0.07	− 0.07 (0.71)	0.9180
Cortisol (18.12, 7139.34)	670	0.03 (0.4021)	0.07	0.00 (0.00)	0.2896	0.07	0.00 (0.00)	0.3026
log(cortisol) (2.90, 8.87)	670	0.03 (0.4021)	0.08	0.25 (0.14)	0.0871	0.07	0.22 (0.15)	0.1274
PSS (0, 37)	1220	0.13 (< 0.0001)	0.08	0.06 (0.02)	< 0.0001	0.09	0.07 (0.02)	< 0.0001
PSQI (0, 21)	1199	0.19 (< 0.0001)	0.12	0.20 (0.03)	< 0.0001	0.12	0.21 (0.03)	< 0.0001
DOM1 (4–20)	1225	− 0.17 (<0.0001)	0.10	− 0.22 (0.03)	< 0.0001	0.10	− 0.23 (0.04)	< 0.0001
DOM2 (4–20)	1224	− 0.06 (0.0404)	0.08	− 0.11 (0.04)	0.0104	0.08	− 0.12 (0.04)	0.0085
DOM3 (4–20)	1222	− 0.04 (0.1689)	0.07	− 0.05 (0.04)	0.2342	0.07	− 0.04 (0.04)	0.2916
DOM4 (7–20)	1225	− 0.14 (<0.0001)	0.09	− 0.25 (0.05)	< 0.0001	0.09	− 0.25 (0.05)	< 0.0001

*PSS* perceived stress scale, *PSQI* Pittsburgh Sleep Quality Index, *DOM1* the physical health domain of the WHOQOL-BREF, *DOM2* the psychological well-being domain of the WHOQOL-BREF, *DOM3* the social relationships domain of the WHOQOL-BREF, *DOM4* the environmental factors domain of the WHOQOL-BREF

^a^Linear regression model is adjusted for distance and province

^b^The slope (SE) standard error corresponds to that of the variable listed in column 1 of the table

^c^Linear regression model is adjusted for distance, province, age, and sex

## Discussion

The current analysis investigated the potential statistical association between aggregate annoyance and health outcomes that were either subjectively reported or objectively measured in the CNHS. Although the associations observed were not as widespread as they were when the analysis was limited to high WTN annoyance (Michaud et al. 2016a), higher aggregate annoyance scores were found to correlate with an increase in diastolic blood pressure, perceived stress (i.e., PSS), rated sleep quality over the previous 30 days (i.e., PSQI scores), physical health, psychological well-being, and environmental factors as measured by the WHOQOL-BREF domains. Annoyance was also higher among participants reporting chronic pain, tinnitus, migraines/headaches, dizziness, and high sleep disturbance at home for any reason over the previous year. When considered collectively, an aggregate annoyance level around 2.5 appeared to separate the group reporting these conditions from those that did not. Average aggregate annoyance dropped below 2.5 in the distance ranges (0.550–1) km in PEI and (1–2) km in ON, from wind turbines.(Michaud et al. 2018) Conditions not related to aggregate annoyance included hair cortisol concentrations, systolic blood pressure, and rated quality of life when assessed with the single standalone question. It should be underscored that the observed associations between aggregate annoyance and health outcomes should not be mistakenly interpreted to mean that annoyance causes adverse health effects (or vice versa). These are statistical observations made from data collected at one point in time with no documented historical records for any of the evaluated outcomes or control for other factors that may impact annoyance or health.

Part of the widespread adoption of *high noise annoyance* as a targeted outcome for community noise in general is that the WHO has quantified the burden of disease associated with it (WHO 2011). No equivalent measure is available to calculate the impact associated with lower magnitudes of annoyance, or when annoyance is directed toward non-noise exposures. High noise annoyance has repeatedly been shown to have a statistical association with elevated long-term average sound levels and other health measures (Niemann et al. 2006; Michaud et al. 2016a). The relationship between elevated sound levels and high noise annoyance may be adequate for transportation noise sources and certain resource activities (e.g., mining) where high noise levels are the principal factor driving community annoyance. A change in high noise annoyance by an equivalent of 6.5% has been suggested as one of the potential measures of a significant noise impact in environmental assessments that are subject to Canadian federal government review (Michaud et al. 2008; Health Canada 2016). However, in situations where multiple variables are driving community annoyance, as appears to be the case with utility scale wind turbines, consideration of only high noise annoyance may undermine other emissions that contribute to overall community annoyance.

As data in this area accumulates, there is no reason why an alternative approach, based upon aggregate annoyance, could not eventually be adopted for situations where multiple source features are known to underscore community annoyance reactions. A mean aggregate annoyance score that could reliably distinguish participants who self-report health effects (or noise complaints) from those who do not could be one of several factors considered by jurisdictions responsible for decisions regarding wind turbine developments. Decisions would have even more support if aggregate annoyance scores could be reliably associated with objectively measured health outcomes. However, the threshold value for acceptable changes and/or levels in aggregate annoyance has not yet been established and some insight may be gained in this regard from future research. Additional research in this area could also assess the perceived valuation attributed to various wind turbine features. For example, aggregate annoyance as an outcome that has some relevance to land-use planning assumes that rated measures of annoyance toward noise, shadow flickers, blinking lights, vibrations, or overall visual impacts represent the attributed impact that people assign to each of these wind turbine features. The assumption is that instructing respondents to recall their exposure *over the previous year* before reporting their annoyance level balances differences between wind turbine features, be that in exposure and/or the level of effort one invests in coping with each.

It should also be underscored that in response to concerns raised during the external peer review of this paper, the association between the non-noise annoyance variables and self-reported and measured health outcomes was evaluated. With the exception of vibration annoyance, which could not be evaluated due to the small sample size, blinking lights, shadow flicker, and visual annoyance were found to be statistically associated with several measures of health, including, but not limited to, migraines, dizziness, tinnitus, chronic pain, sleep disturbance, perceived stress, quality of life measures, lodging a WTN-related complaint, and measured diastolic blood pressure. Although these annoyance-specific associations with various health measures lend support to actions that may rely on an aggregate annoyance measure, it would be of interest to compare findings from stated choice experiments to results based on rated annoyance. Stated choice studies can estimate the value assigned to each wind turbine feature using a willingness to pay/accept model similar to that presented by Thanos Wardman and Bristow for aircraft noise valuation (Thanos et al. 2011). Finally, although aggregate annoyance has been presented as a construct that reflects a more complete measure of community annoyance toward wind turbines (Michaud et al. 2018), additional research could investigate indirect factors for their potential contribution to community annoyance (e.g., perceived impacts on property value, electricity costs, and wildlife). Similarly, perceived benefits to the environment could be evaluated as nullifying rated annoyance toward any given wind turbine feature.

As this area of research matures, new findings may identify an aggregate annoyance value that corresponds to a threshold for community acceptability. Although individual exposure response relationships with a clear point of departure in the curve can inform policy decisions, their interpretation can be complicated when separate exposure response functions differ in the overall prevalence of annoyance or when their pattern of change is inconsistent across multiple exposure categories. These issues can be addressed, in part, with an exposure response based upon an aggregate annoyance construct.
